# Multiple Wireless SAW Interrogators Based on Software-Defined Radios

**DOI:** 10.3390/mi17101209

**Published:** 2026-10-21

**Authors:** Jianfei Jiang, Yangfan Liang, Hong Zhang

**Affiliations:** 1 College of Biomedical Engineering & Instrument Science, Zhejiang University, Hangzhou 310027, China; jiangjianfei@zju.edu.cn; 2 Sino-European Joint Lab for Health Information Processing and Applications, Jiaxing University, Jiaxing 314001, China; 3 College of Information Science & Electronic Engineering, Zhejiang University, Hangzhou 310027, China

**Keywords:** wireless passive sensor, wide-band interrogation, SAW, USRP, GNU Radio

## Abstract

Multiple SAW sensors working in separate frequency bands can encode multiple measurands in the frequency domain during a single interrogation. In such applications, software-defined radio is the most convenient platform to transceive wideband RF signals and process them in digital mode. This paper introduces a novel SDR platform that can interrogate a large number of resonant SAW sensors simultaneously. These sensors can be distributed in a frequency range from 70 MHz to 6 GHz, with an instantaneous bandwidth of 56 MHz. The 56 MHz wideband excitation signal is transmitted and received without frequency sweeping, so sensors operating at different resonant frequencies can be stimulated simultaneously, which is critical for accurate frequency-based measurement in multi-sensor applications. Five-sensor interrogation is described as an example, with an interrogation period of 15.12 μs. As the transceiver operates in time-division mode, the delay before reception and the listen-window width are designed to be parameter-adjustable to fit diverse applications, thereby improving system performance. The described SDR platform is based on USRP-B210 hardware and GNU Radio software. The USRP-B210 operates at its maximum sampling rate of 61.44 MHz with a bandwidth of 56 MHz. For decay-time-limited resonant sensors, the maximum sampling rate captures the most samples of the response signal, resulting in improved SNR performance.

## 1. Introduction

Wireless passive Surface Acoustic Wave (SAW) sensors have been extensively utilized in various industrial and remote sensing applications, including the monitoring of temperature, humidity, vibration, pressure, and torque [1,2,3,4,5,6,7,8,9,10,11]. Due to their passive nature, these sensors require a radio frequency (RF) transceiver [12] to transmit an excitation signal and capture the subsequent response from the SAW devices. The received responses are then processed in either the time or frequency domain to extract the physical parameters. Conventionally, Frequency Modulated Continuous Wave (FMCW) or Frequency Stepped Continuous Wave (FSCW) signals are employed as the primary excitation signals in these interrogation systems [13]. In complex environmental monitoring, traditional hardware is insufficient in terms of bandwidth and flexibility for excitation. So, it is difficult to meet the synchronous detection requirements of multi-band and distributed SAW sensors.

**Figure 1:**
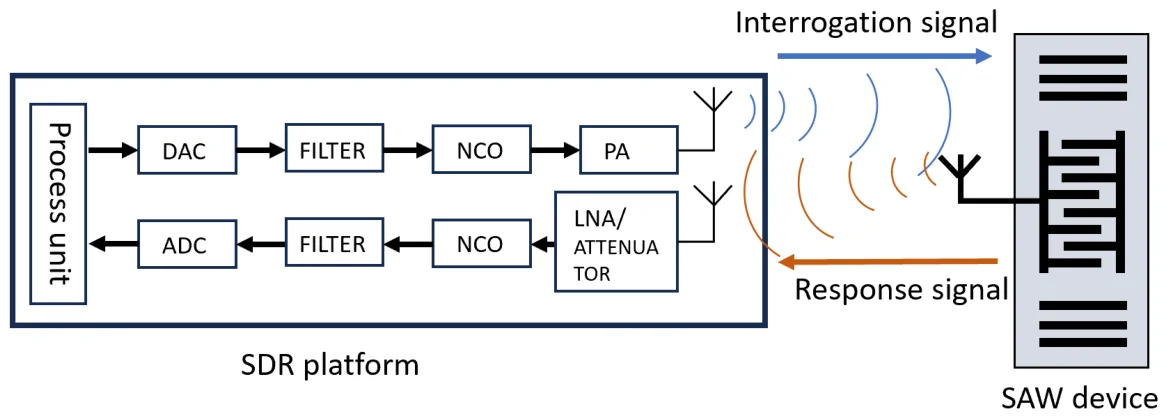
A general SDR platform.The software running in the control unit and process unit handlesbase signal processing, and the hardware is designed to cover the application range and is controlled by the software.

Software Defined Radio (SDR) offers a highly flexible architecture capable of overcoming the rigid limitations of traditional RF hardware [14]. In an SDR system, the baseband signal is generated digitally via software, converted to an analog signal by a Digital-to-Analog Converter (DAC), and subsequently transmitted through an adaptable analog chain [15]. The receiving process follows a reverse routine, enabling complex digital signal processing on the host computer. Figure 1 illustrates the general architecture of an SDR platform adapted for SAW interrogation applications.

The USRP-B210, developed by ETTUS Research [16] (Santa Clara, CA, USA), is a high-performance SDR platform operating over a wide frequency range from 70 MHz to 6 GHz with a maximum instantaneous bandwidth of 56 MHz. The B210 offers a maximum sampling rate of 61.44 MHz. These features support wideband SAW sensor networks. The device is compatible with GNU Radio, an open-source software platform. GNU Radio provides digital signal processing functions. It also allows flexible parameter configuration and offers an intuitive user interface.

However, in the case of multiple SAW sensor queries, the existing SDR platforms still encounter bottlenecks [17,18]. At the maximum sampling rate (61.44 MHz), the data stream is extremely likely to exceed the USB throughput limit. This will cause transmission between the host and the SDR to be blocked and result in severe data loss during the reception window period. Second, conventional frequency-sweeping signals (such as Chirp) are inadequate for simultaneous wideband interrogation. Because the sweep occurs over time, sensors with different resonant frequencies are excited sequentially rather than instantly, introducing systematic frequency measurement biases and missing critical decay responses for time-division sensors.

To resolve these issues, this paper introduces a novel SDR-based interrogation platform that can simultaneously excite and process multiple resonant SAW sensors spread across a wide frequency range. We implemented modifications to the FPGA of the USRP-B210 to include a customized FIFO buffering strategy and an on-board signal generator, completely bypassing the USB throughput bottleneck. Furthermore, we replaced the traditional Chirp signal with a Pseudo-Random Noise (PRN) excitation signal, which fully occupies the 56 MHz bandwidth instantaneously without sweeping. This design ensures that all sensors in the frequency band are excited simultaneously, which is critical for accurate frequency-based measurements of resonant SAW sensors.

The organization of this paper is as follows: Section 2 presents related works in SAW interrogation and SDR applications. Section 3 presents the theoretical background. Section 4 details the specific hardware and FPGA modifications made to the USRP-B210. Section 5 analyzes and compares different excitation signal types, justifying the choice of PRN over Chirp. Section 6 presents the experimental setup and discusses the interrogation results of multiple SAW sensors. Finally, Section 7 concludes the paper.

## 2. Related Works

### 2.1. Traditional SAW Sensor Interrogation Techniques

Wireless passive surface acoustic wave (SAW) sensors are commonly used in harsh environments. They can effectively monitor physical parameters such as temperature, pressure, and strain [1,2,3,4,5,6,7,8,9,10,11]. Traditional detection systems are based on radar architecture and mainly use FMCW or FSCW signals [13]. These systems rely on pure hardware design and have high accuracy and good performance when monitoring a single sensor. However, traditional systems lack flexibility. Their hardware is usually customized for specific frequency bands. When adapting or upgrading multi-frequency band sensors, the cost is high and the process is cumbersome [19,20]. Moreover, the synchronization processing of multiple sensors is difficult. This requires complex analog circuits and frequent frequency scanning. These factors limit the detection speed and reduce the overall agility of the system.

### 2.2. SDR-Based Interrogation Systems

Traditional hardware has rigid limitations. To overcome this drawback, software-defined radio (SDR) has become an important platform for SAW sensor detection [14]. SDR shifts the heavy tasks such as signal generation and processing from dedicated analog hardware to the digital domain. This transformation brings extremely high flexibility to the system. Now, the working frequency, bandwidth, and modulation mode of the system can all be dynamically adjusted [21]. Several studies have validated the feasibility of USRP (Universal Software Radio Peripheral) platforms for this purpose. For instance, Humphries et al. proposed an interrogation device based on the USRP-B200 platform in 2015 [17]. By modifying the FPGA function to fit the interrogation routine, they achieved an interrogation bandwidth of 35 MHz using a chirp signal, with a single interrogation cycle of approximately 265 μs. In another application based on the USRP-N200, an interrogation bandwidth of 10 MHz and a single cycle of 12.5 μs were reported, where the received response signal was processed using time correlation for delay-time measurement [18].

A quantitative comparison between the proposed system and previously reported USRP-based SAW interrogation platforms is presented in Table 1.

The key distinctions of the proposed system from previous work include: (1) full utilization of the 56 MHz instantaneous bandwidth of the USRP-B210, compared to 35 MHz (B200) and 10 MHz (N200); (2) simultaneous interrogation of five sensors in a single cycle, whereas previous systems interrogated one sensor per cycle; (3) replacement of Chirp with PRN excitation, eliminating sweep-induced timing errors; and (4) implementation of on-board FIFO buffering and block-RAM signal generation in the FPGA, completely bypassing the USB throughput bottleneck.

### 2.3. Limitations in Current SDR Approaches and Signal Types

SDR technology has several advantages. However, its hardware capability has not been fully utilized in existing studies, especially on the USRP-B210 for multi-sensor applications [22]. The USB connection between the SDR device and the host computer is still a performance bottleneck. It limits the data transfer rate. This constraint lowers the effective sampling rate and reduces the interrogation bandwidth. Therefore, only a limited number of sensors can be interrogated simultaneously.

For resonant SAW sensors, the excitation signal is another important factor. Existing SDR applications predominantly rely on Chirp signals, which sweep the frequency over a limited range [17]. As SAW sensors operate in a time-division mode, the frequency sweeping nature of Chirp signals means that sensors with different resonant frequencies are excited at different moments within the sweep cycle [23,24,25]. This temporal discrepancy often causes the receiver to miss critical response data if the sensor’s decay time is shorter than the sweeping period. Additionally, environmental variations cause resonant frequencies to shift, meaning the sweeping mode inherently introduces systematic frequency measurement biases that require complex post-processing to correct.

### 2.4. Motivation of This Work

To address the aforementioned challenges, this paper proposes an enhanced SDR platform. This platform fully utilizes the 56 MHz complete bandwidth of the USRP-B210. We introduced pseudo-random noise (PRN) as the excitation signal. This signal can occupy the entire working bandwidth instantly without the need for frequency scanning. We implemented a customized FIFO buffer in the FPGA. The buffer eliminates the systematic frequency measurement biases introduced by Chirp signals. It also prevents data loss when the sampling rate reaches 61.44 MHz. This allows the system to interrogate many distributed resonant SAW sensors simultaneously.

## 3. Theoretical Background

This section describes the principles behind the proposed interrogation system. It begins with the operating mechanism of passive wireless SAW sensors, including piezoelectric energy conversion and acoustic delay. These characteristics determine the use of time-division multiplexing (TDM) in the SDR interrogator. They also influence the FPGA architecture and timing design described in the following sections.

### 3.1. Operating Principle of Wireless Passive SAW Sensors

Wireless passive SAW sensors do not require an internal power source [26,27]. They harvest energy from the radio frequency signal transmitted by the interrogator. Each sensor consists of an antenna, an interdigital transducer (IDT), and a reflector array fabricated on a piezoelectric substrate [28]. After the excitation signal is received by the antenna, the IDT generates a surface acoustic wave through the inverse piezoelectric effect [29]. The wave travels across the substrate and is reflected by the reflector array [26]. Environmental variations, such as temperature changes, modify the acoustic velocity and the substrate dimensions [30]. These changes appear as shifts in resonant frequency or propagation delay. The returning acoustic wave is converted into a radio frequency signal by the IDT through the direct piezoelectric effect before being received by the interrogator. The response exists for only a few microseconds and decays rapidly after reflection. Its short duration and propagation delay determine the timing requirements of the receiver [13].

### 3.2. Time-Division Interrogation Strategy in SDR

The excitation signal has much higher power than the signal returned by the sensor. If the transmitter and receiver operate at the same time, the transmitted signal can leak into the receiving path, resulting in receiver saturation or even damage to the RF front end [31]. Therefore, the interrogation system adopts a half-duplex time-division multiplexing (TDM) scheme. An interrogation cycle begins with transmission of a broadband excitation signal. After transmission, the system waits briefly for the residual energy in the antenna and RF circuits to decay. The receiver is then switched on to capture the weak echo from the sensor [13]. Reliable operation depends on accurate timing control. The transition between transmission and reception must be carefully scheduled to avoid interference and to ensure complete signal acquisition. These timing requirements are reflected in the FPGA implementation described later [14].

## 4. Modification to USRP

### 4.1. Throughput Bottleneck and Time-Division Interrogation Strategy

The USRP-B210 utilizes the USRP Hardware Driver (UHD) as its standard host–device interface. Under default factory UHD configurations, continuous real-time streaming over the USB 3.0 bus is constrained to a maximum sustained data rate. This bandwidth limitation is inherently insufficient to support the continuous, un-dropped transfer of raw I/Q samples when the system operates at its peak sampling rate of 61.44 MHz.

Fortunately, because wireless passive Surface Acoustic Wave (SAW) sensors operate in a time-division mode, the interrogation process does not require simultaneous transmission and reception. The effective receiving window is strictly bounded to the short duration of the sensor’s acoustic decay response. Capitalizing on this operational characteristic, the hardware project within the FPGA of the USRP-B210 was modified. By introducing an on-board temporary buffering mechanism, high-rate analog-to-digital converter (ADC) samples collected during the response window can be buffered locally and subsequently transferred to the host computer without any data dropping, thereby enabling full-bandwidth interrogation.

### 4.2. Custom FIFO Buffer Implementation

**Figure 2:**
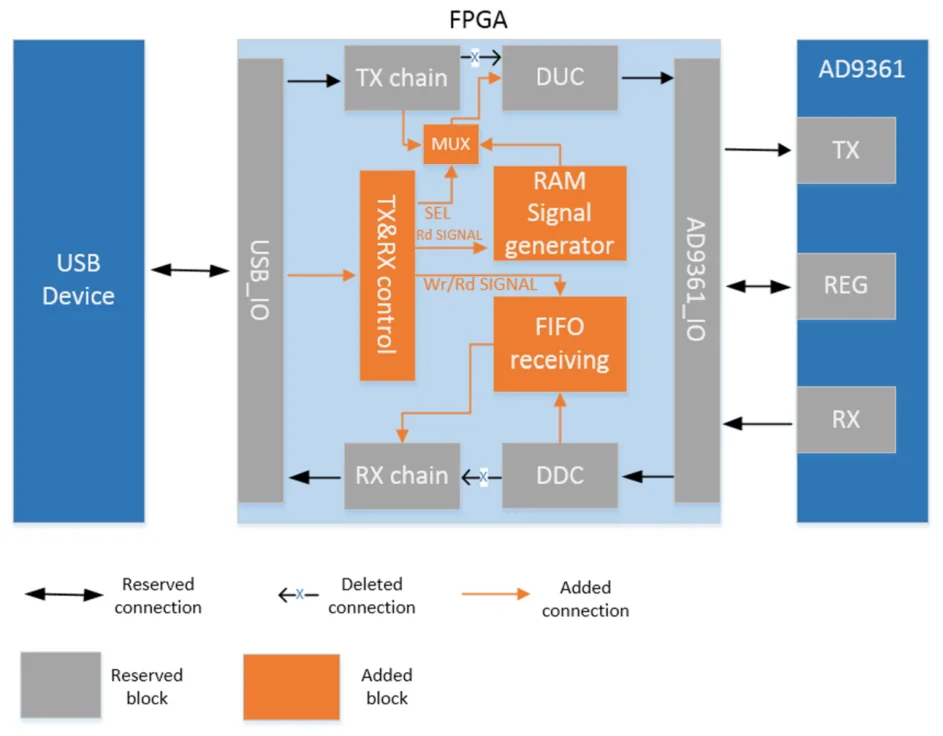
FPGA function diagram after modification.

To prevent data loss at the 61.44 MHz sampling rate, a customized first-in, first-out (FIFO) receiving buffer was integrated into the primitive hardware architecture, as illustrated in the block diagram in Figure 2. The custom FIFO core is implemented with a native depth of 1024 words, and its read and write ports are managed by a centralized transmission and reception control (TX & RX Control) block.

Importantly, because data can be pushed into and read out of the FIFO concurrently, the maximum packet size that can be buffered and transferred to the host in its entirety is not strictly restricted by the physical 1024-word depth. As long as the host read transaction remains active within the sampling window, the effective maximum data packet size is greatly enhanced, significantly breaking through the original physical limitations of the FPGA hardware.

The custom FIFO is implemented using the Xilinx LogiCORE IP FIFO Generator (v9.3, AMD, Santa Clara, CA, USA), configured for a native width of 32 bits (16-bit I + 16-bit Q) and a depth of 1024 words. The FIFO write port operates in the ADC sample clock domain (61.44 MHz), while the read port operates in the USB clock domain (60 MHz), with the FIFO safely handling the clock domain crossing. The TX and RX Control block monitors the FIFO fill level through the almost_full and almost_empty threshold signals. When the fill level reaches 75%, the control block initiates a host transfer request, preventing overflow while maximizing throughput.

Under this specific configuration, the stability of data transmission is significantly increased. Without losing any data, the maximum data packet size can reach 4096 samples when the host-side read throughput is maintained at a sustained rate. At a sustained USB 3.0 read throughput of approximately 30 MB/s, the maximum burst length before FIFO overflow is 4096 samples (equivalent to 66.7 μs at 61.44 MHz), which far exceeds the 512-sample receiving window used in the current implementation. Especially at a 61.44 MHz sampling rate, when the query response time of the broadband SAW sensor exceeds 1024 samples, the FIFO provides critical cache support.

### 4.3. On-Board Excitation Signal Generator via Block RAM

In addition to the receiving buffer, we also added a RAM module to the FPGA architecture. This module serves as an independent on-board excitation signal generator. In traditional SDR systems, the host needs to continuously transmit streaming excitation waveforms to the device. This would consume a significant amount of USB throughput. To eliminate this bandwidth overhead, this scheme uses MATLAB (R2018a, MathWorks, Natick, MA, USA) to offline synthesize wideband excitation signals and uses them to initialize the on-board RAM.

In the active interrogation loop, this RAM acts as a local signal source in each cycle. Since the signal generation is fully running inside the FPGA, the transmission chain (TX chain) does not consume any USB bus bandwidth during operation. This design significantly improves the overall interrogation efficiency of the system and reserves the largest bus bandwidth for the receiving chain (RX chain).

### 4.4. Interrogation Control Logic and Precise Timing Execution

The TX & RX control module is the core coordinating unit of the modified hardware architecture. This module continuously analyzes the instructions from the host, sequentially executes the query process, and achieves continuous monitoring through cyclic operation. The entire query process is strictly divided into three deterministic steps, i.e., sending, delay, and receiving. The control module implements a 3-state finite state machine (TX → DELAY → RX) with hardware counter-based timing for each state. The TX state duration is configurable from 1 to 256 clock cycles (default: 128 cycles for the 128-sample PRN), the DELAY state is configurable from 1 to 512 clock cycles (default: 150 cycles), and the RX state is configurable from 1 to 4096 clock cycles (default: 512 cycles). The state transitions are triggered by counter overflow, ensuring deterministic timing independent of host-side software latency.

For resonant SAW sensors, accurate frequency measurement requires that the echo signal be captured with consistent timing and phase conditions across interrogation cycles. Maintaining strict time-domain consistency is therefore of utmost importance. To meet this requirement, independent hardware counters are integrated in each operation step. Dedicated counters control the duration of each stage with a timing resolution of one FPGA clock cycle. Since all timing signals are generated inside the FPGA, host-side scheduling jitter does not affect system operation.

## 5. Analysis of Excitation Signal Types

The excitation signal has a direct impact on the performance of the SDR interrogation system. Its characteristics affect both the available bandwidth and the detection accuracy. This section evaluates several broadband excitation signals for multi-sensor interrogation and explains the selection of the proposed signal. Firstly, we explore the characteristics and limitations of the traditional chirp signal. We focus on analyzing the negative impacts it has on timing synchronization and data acquisition within a strict half-duplex framework. Then, we introduce pseudo-random noise as a more robust alternative. PRN has unique instantaneous spectral distribution characteristics. This feature enables it to effectively overcome the time sweeping problem brought about by the chirp signal, thereby achieving true multi-sensor synchronous excitation.

### 5.1. Characteristics and Limitations of Chirp Signals

The chirp signal has been proven to have a significant effect on the detection of frequency-based SAW sensors. A universal frequency-modulated signal can be defined by its instantaneous frequency. The time-domain waveform S(t) can be expressed as

(1) S(t)=cos(2πf(t)t)

For a typical linear Chirp signal, the frequency increases or decreases linearly over time. The corresponding waveform is represented as:

(2) $$S\left(t\right)=\cos\left(2\pi (f_{0}+kt)t\right)$$

**Figure 3:**
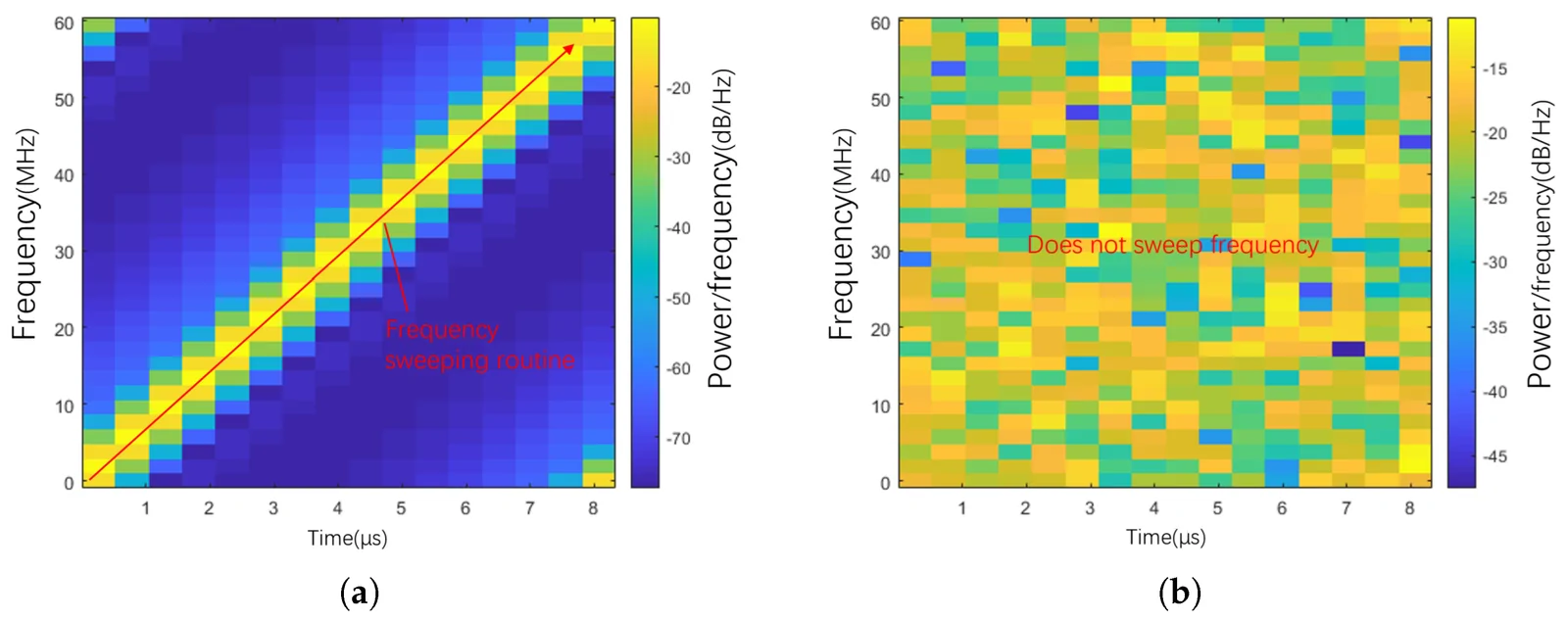
Spectrogram comparison of Chirp and PRN signal. (**a**) Chirp signal sweeps from start time to end time. (**b**) PRN signal does not work in sweeping mode.

Here, $f_{0}$ represents the starting frequency, and *k* represents the chirp rate. Equation (2) indicates that the chirp signal will sequentially sweep through the entire frequency range over a specified period of time (as shown in Figure 3a). However, in half-duplex time division operation mode, the use of chirp signals poses severe challenges. In this mode, the system must completely finish the signal transmission before it can start receiving the signal.

This “send first, then receive” mechanism leads to serious loss of response data. When the characteristic frequency of the sensor is close to the start of the sweep frequency, it will be prematurely excited in the initial stage of transmission. But the receiver must wait until the entire sweep cycle (for example, 8 μs) is over before starting to sample. If the signal attenuation time of the sensor is short (for example, 8.3 μs), its high-energy early response will be exhausted during the waiting period. In severe cases, the system may even fail to capture any useful data.

Furthermore, environmental changes such as temperature drift can cause the resonant frequency of the SAW sensor to dynamically shift. Since the chirp signal excites different frequencies at different times, this frequency shift directly converts into systematic frequency measurement errors due to the time-dependent nature of the chirp excitation. Eliminating such errors usually requires the introduction of complex and time-consuming post-processing algorithms.

### 5.2. Advantages of Pseudo-Random Noise

To circumvent the inherent limitations of frequency-sweeping signals, Pseudo-Random Noise is adopted as a superior alternative for wideband excitation. Figure 3b illustrates the time–frequency domain representation of the PRN signal.

Unlike a Chirp signal, the PRN signal does not sweep; instead, its spectral energy occupies the entire designated bandwidth instantaneously and consistently at every moment during transmission. This broad and instantaneous spectral distribution ensures that all sensors within the frequency band are excited simultaneously. Consequently, it eliminates the timing discrepancy between sensors with different characteristic frequencies, mitigates the systemic errors in delay-time measurements, and maximizes the capture of the sensors’ effective decay responses within the limited receiving window.

### 5.3. Mathematical Model of PRN-Based SAW Echo Measurement

To provide a rigorous theoretical foundation for the performance improvement of PRN over Chirp, a mathematical model of the SAW sensor echo measurement under PRN excitation is established in this section. The SAW sensors used in this work are of the resonant type, where the measurand (temperature) modulates the sensor’s resonant frequency $f_{0}$.

#### 5.3.1. Signal Model

The received signal r(t) is modeled as the convolution of the PRN excitation signal p(t) with the sensor impulse response h(t) in the presence of additive white Gaussian noise n(t):

(3) r(t)=p(t)∗h(t)+n(t)

The impulse response of a resonant-type SAW sensor is a damped oscillation at the sensor’s resonant frequency $f_{0}$:

(4) $$h\left(t\right)=\alpha \cdot e^{-t/\tau_{d}}\cdot e^{j2\pi f_{0}t}\cdot u\left(t\right)$$

where α is the amplitude factor determined by the insertion loss and antenna coupling, $\tau_{d}$ is the decay time constant of the resonator (related to the quality factor *Q* by $\tau_{d}=Q/\left(\pi f_{0}\right)$), $f_{0}$ is the resonant frequency of the sensor, and u(t) is the unit step function. The environmental measurand (e.g., temperature) modulates $f_{0}$, and the sensor response is characterized primarily by its resonant frequency rather than a propagation delay. A fixed propagation delay $\tau_{0}$ due to the acoustic path and wireless link is also present but is not the measured quantity.

#### 5.3.2. SNR Analysis

For PRN excitation, the processing gain $G_{p}$ achieved through cross-correlation is:

(5) $$G_{p}=10\cdot \log_{10}\left(B\cdot T\right)\;\left[\mathrm{dB}\right]$$

where *B* is the instantaneous bandwidth (56 MHz) and *T* is the PRN sequence duration. For a 128-sample PRN at 61.44 MHz sampling rate, *T* = 2.083 μs, yielding:

(6) $$G_{p}=10\cdot \log_{10}\left(56\times 10^{6}\times 2.083\times 10^{-6}\right)\approx 20.7\;\left[\mathrm{dB}\right]$$

For Chirp excitation with the same bandwidth and duration, the processing gain is theoretically identical ($G_{c}=B\cdot T$), but the effective SNR is degraded by the timing mismatch loss $L_{t}$ caused by the sequential excitation nature of Chirp:

(7) $${\mathrm{SNR}}_{\mathrm{PRN}}={\mathrm{SNR}}_{0}+G_{p}$$

(8) $${\mathrm{SNR}}_{\mathrm{Chirp}}={\mathrm{SNR}}_{0}+G_{c}-L_{t}$$

where ${\mathrm{SNR}}_{0}$ is the base SNR and $L_{t}$ is the timing mismatch loss caused by the sequential excitation nature of Chirp in half-duplex mode. $L_{t}$ varies depending on the sensor’s position within the frequency sweep: sensors excited near the beginning of the sweep lose more echo energy before the receiver is enabled, while those excited near the end lose less. For a 2.083 μs sweep (128 samples at 61.44 MSPS) covering 35 MHz bandwidth, and sensors with typical decay time constants of 1.7–2.3 μs, $L_{t}$ ranges from approximately 2 dB (for the latest-excited sensor near 468.5 MHz) to about 7 dB (for the earliest-excited sensor near 445.5 MHz). This is because the echo energy decays exponentially during the wait between excitation and receiver activation. Even with a relatively short 2.083 μs sweep, the earliest-excited sensor retains only about 22% of its echo energy by the time reception begins, while the latest-excited sensor retains about 64%. This demonstrates a clear SNR advantage of PRN over Chirp for resonant SAW sensors operated in half-duplex mode, with the advantage being most pronounced for sensors near the lower end of the frequency band.

#### 5.3.3. Measurement Error Analysis

For resonant SAW sensors, the primary measurand is the resonant frequency $f_{0}$. The frequency estimation error for a sinusoidal signal in white noise is given by the Cramér–Rao Lower Bound (CRLB):

(9) $$\sigma_{f_{0}}\geq \sqrt{\frac{3\cdot f_{s}^{2}}{2\pi^{2}\cdot \mathrm{SNR}\cdot N\cdot \left(N^{2}-1\right)}}$$

where $f_{s}$ is the sampling rate, *N* is the number of observation samples, and SNR is the signal-to-noise ratio. For the system parameters ($f_{s}=61.44$ MHz, N=512, SNR=20 dB after processing gain), the theoretical minimum frequency estimation error is approximately:

(10) $$\sigma_{f_{0}}\approx \sqrt{\frac{3\cdot {\left(61.44\times 10^{6}\right)}^{2}}{2\pi^{2}\cdot 100\cdot 512\cdot \left(512^{2}-1\right)}}\approx 0.21\;\mathrm{kHz}$$

This sub-kHz theoretical lower bound indicates that the kHz-level measurement precision achieved in practice can be further improved by more advanced estimation algorithms, such as the parabolic peak interpolation described in Section 6.4.

## 6. Experiment and Result

### 6.1. Experimental Setup

**Figure 4:**
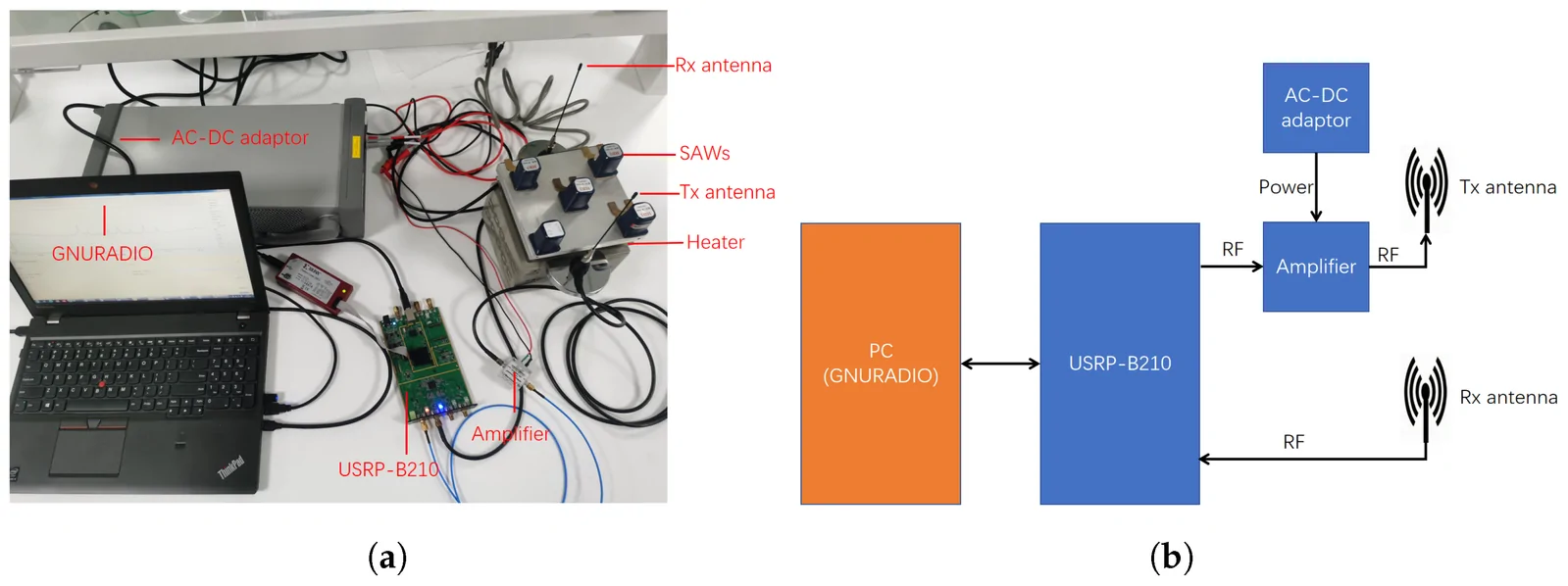
Experiment setup. (**a**) Equipment and location. (**b**) Function block diagram.

To demonstrate the capabilities of the proposed SDR equipment, an interrogation experiment involving five SAW sensors was conducted. Figure 4 presents the experimental setup. The transmitter and receiver are connected to separate antennas, which reduces direct coupling between the two paths. Five SAW sensors are mounted on an electric heater (ZB1515JR, Zhejiang Huaqi Zhengbang Automation Technology Co., Ltd., Lishui, China) during the experiments. This experimental setup was specifically designed to monitor the temperature variations in the heater in real time.

The five SAW sensors used in this experiment are of the resonant type, which is a widely employed configuration for wireless passive SAW temperature sensing applications. Each sensor consists of an interdigital transducer (IDT) for electro-acoustic conversion and two reflector gratings positioned on either side of the IDT, forming a one-dimensional resonant cavity. The resonant-type architecture was chosen because it produces a high-*Q* frequency response, enabling precise frequency-shift-based temperature measurements.

All five sensors are fabricated on Y-cut X-propagating quartz substrates, which exhibit a positive temperature coefficient of frequency (TCF) of approximately 22 ppm/°C at the design frequencies. This quartz substrate was selected for its good temperature stability, low acoustic propagation loss, and well-established fabrication processes. The substrate dimensions are 4.0 mm × 1.5 mm × 0.5 mm for each sensor. The IDT was fabricated with a finger period of 7.2 μm, corresponding to an acoustic wavelength of approximately 7.2 μm at the design frequency. Each sensor was custom-fabricated in-house using standard photolithography and metal deposition techniques. The five sensors were designed to operate at distinct characteristic frequencies in the range of 445–469 MHz to enable frequency-division multiplexing within the 56 MHz instantaneous bandwidth of the USRP-B210. Each sensor is wirelessly coupled to the interrogation system through a planar loop antenna with a bandwidth of approximately 80 MHz centered at 457 MHz, ensuring coverage of all five sensor frequencies.

### 6.2. GNU Radio Implementation and Interrogation Workflow

**Figure 5:**
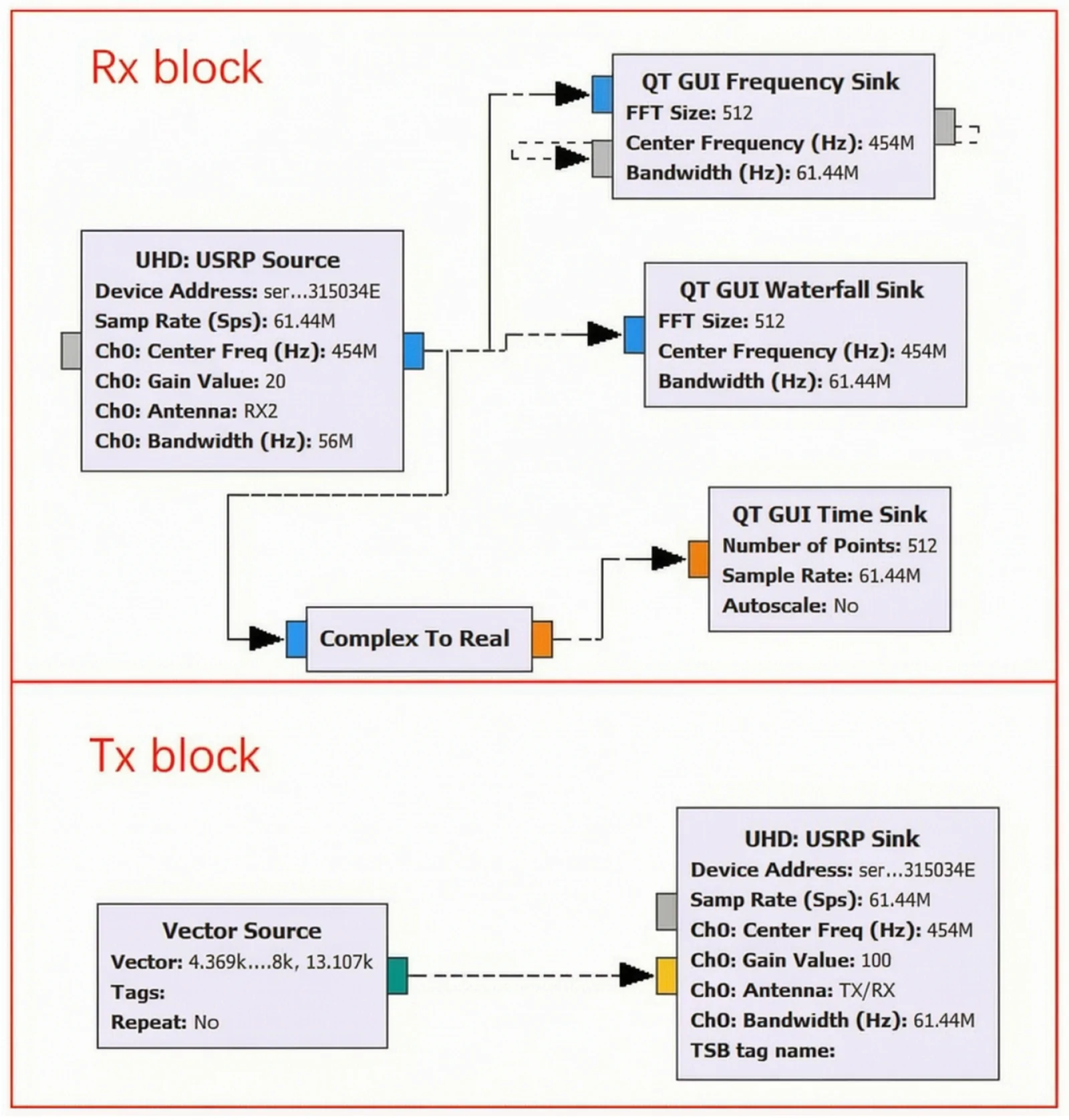
GNU Radio function design block.

GNU Radio (GNU Radio Companion 3.7.13.5) was utilized to initialize the interrogation process, configure the transmission (TX) and reception (RX) center radio frequencies, set the TX and RX gains, and select the appropriate hardware channels. A Qt-based graphical user interface (GUI) toolbox was employed to receive the SAW response signals and visualize them through spectrograms and waveform diagrams. Additionally, the software allows the received data to be saved as local files for offline post-processing in MATLAB. Figure 5 illustrates the experimental setup alongside the GNU Radio block diagram.

**Figure 6:**
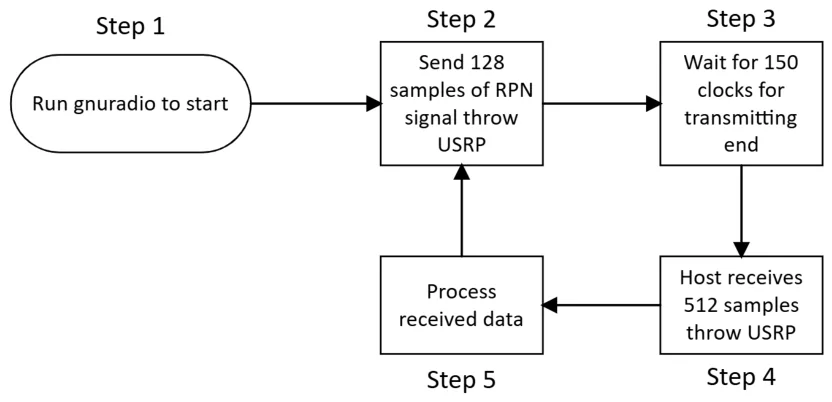
Interrogation work flow.

Figure 6 shows the interrogation procedure. After the GNU Radio flow graph is launched, the USRP-B210 transmits a 128-sample PRN excitation signal. The FPGA inserts a guard interval of 150 clock cycles before enabling the receiver, allowing residual transmission energy to decay. The receiver then acquires 512 response samples from the SAW sensor. After buffering in the FPGA, the samples are transferred to the host through the USB interface for signal processing. The processed results are displayed in the GNU Radio interface. After this, the system automatically repeats steps 2 to 5. This cycle continues to run until the user manually terminates the GNU Radio program.

### 6.3. Interrogation Results and Analysis

**Figure 7:**
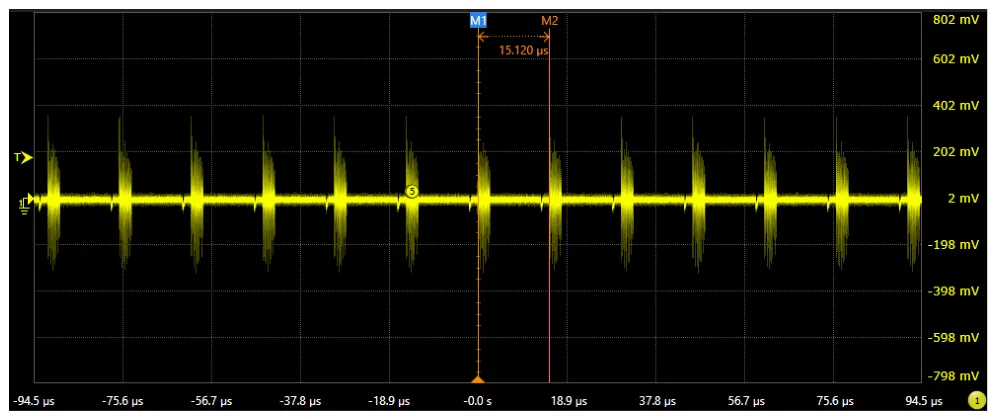
Capture the transmitting signals by a digital oscilloscope. The measured transmitting period is 15.12 μs, which represents the interrogation period.

According to the workflow definition, a single internal loop requires a total of 790 clock cycles. At a sampling rate of 61.44 MHz, the corresponding theoretical time is 12.86 μs. However, during actual operation, data transmission at the host end and algorithm processing introduce additional system delays. Therefore, a digital oscilloscope (Infiniium V-Series DSAV134A, Keysight Technologies, Santa Clara, CA, USA) was used to measure the actual interval between adjacent data transmissions. The measured results show that the actual query loop time of the system is 15.12 μs. Figure 7 visually presents the captured periodic transmission signals.

**Figure 8:**
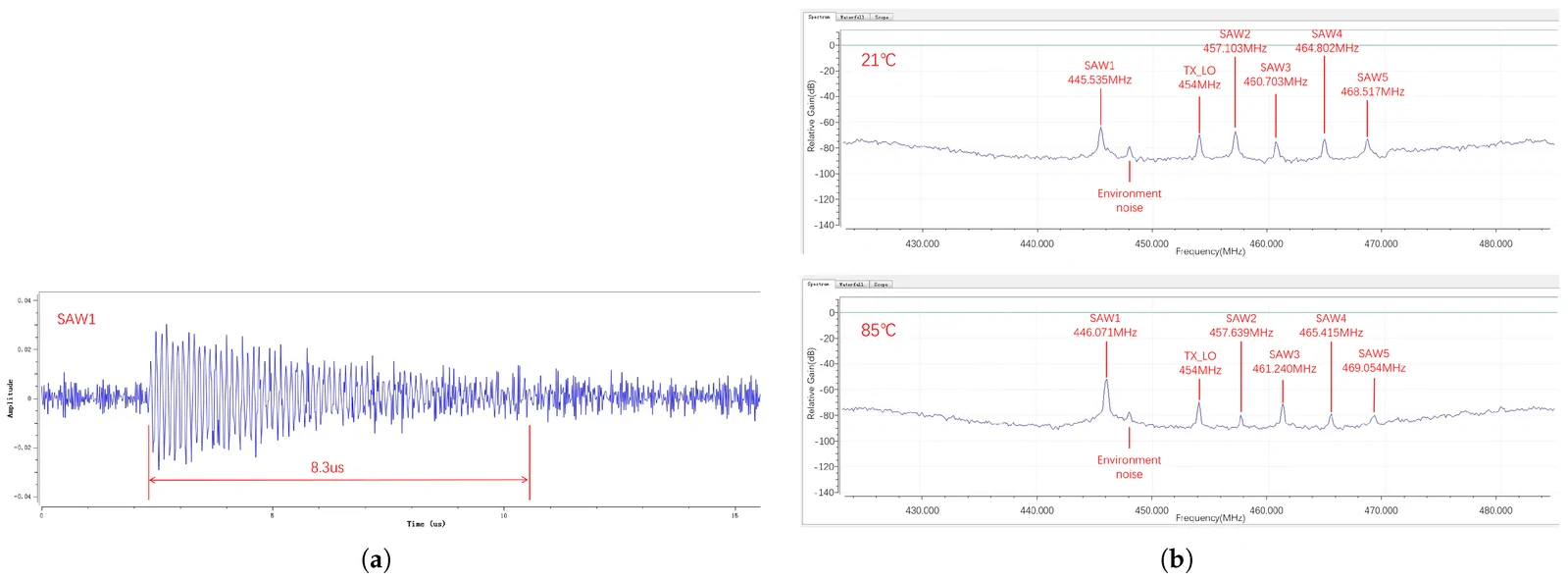
(**a**) Received signal waveform from single SAW. (**b**) Frequencymeasurement result at 21 °C and 85 °C.

Figure 8a displays the received signal waveform from a single SAW sensor. The measured duration of the sensor’s decay response is 8.3 μs. Therefore, allocating 512 samples at a 61.44 MHz sampling rate (which equates to 8.33 μs) provides an adequate window to fully capture the essential response signal without truncation. Figure 8b presents the frequency measurement results at two different heater temperatures: 21 °C and 85 °C. The detailed frequency shifts for all five SAW sensors are recorded in Table 2. As the heater’s temperature increased from 21 °C to 85 °C, the characteristic frequencies of the five sensors varied independently but exhibited a similar overall shifting trend. The measured temperature coefficients of frequency (TCF) for the five sensors range from approximately 18 to 21 ppm/°C, which is consistent with the nominal TCF of Y-cut X-propagating quartz (approximately 22 ppm/°C) considering fabrication tolerances and measurement uncertainties.

### 6.4. Signal Processing for PRN Response Extraction

The received PRN response signal is processed through a two-stage pipeline to extract the resonant frequency of each SAW sensor.

#### 6.4.1. Echo Localization via Cross-Correlation

The received echo signal is first localized in the time domain using cross-correlation between the received signal r(t) and a stored reference copy of the transmitted PRN signal p(t). The cross-correlation function is computed as:

(11) $$R_{rp}\left(\tau \right)=\int r\left(t\right)\cdot p^{*}\left(t-\tau \right)\;dt$$

where $p^{*}\left(t\right)$ denotes the complex conjugate of p(t). The envelope peak of $R_{rp}\left(\tau \right)$ indicates the arrival time of the sensor echo, which is used to determine the appropriate time window for subsequent frequency analysis. Because the PRN signal has an impulsive autocorrelation function, the echo can be localized with high temporal precision. The cross-correlation is performed in the frequency domain using the FFT for computational efficiency:

(12) $$R_{rp}\left(\tau \right)=\mathrm{IFFT}\left\{\mathrm{FFT}\left[r\left(t\right)\right]\cdot \mathrm{conj}\left(\mathrm{FFT}[p(t)]\right)\right\}$$

#### 6.4.2. Resonant Frequency Extraction via FFT

After the echo signal is localized in the time domain using the cross-correlation peak, its resonant frequency is extracted by computing the Fast Fourier Transform (FFT) of the time-domain echo segment. The raw FFT frequency resolution is determined by the sampling rate and the number of time-domain samples:

(13) $$\Delta f_{\mathrm{raw}}=\frac{f_{s}}{N_{\mathrm{time}}}=\frac{61.44\;\mathrm{MHz}}{512}=120\;\mathrm{kHz}$$

where $f_{s}$ is the sampling rate and $N_{\mathrm{time}}=512$ is the number of time-domain samples in the receiving window. This raw resolution of 120 kHz is sufficient to distinguish the five SAW sensors (minimum frequency spacing: 3.6 MHz, corresponding to 30 FFT bins) and to resolve the frequency shifts (∼0.5 MHz) observed in the temperature monitoring experiment.

To achieve sub-bin frequency precision (required for the kHz-level measurement accuracy reported in Table 2), zero-padding is applied to the 512-sample echo segment before FFT computation. The time-domain samples are zero-padded to $N_{\mathrm{FFT}}=4096$ points, which reduces the FFT bin spacing to:

(14) $$\Delta f_{\mathrm{FFT}}=\frac{f_{s}}{N_{\mathrm{FFT}}}=\frac{61.44\;\mathrm{MHz}}{4096}=15\;\mathrm{kHz}$$

Additionally, a parabolic peak interpolation algorithm is applied to the three highest-magnitude FFT bins around each spectral peak, further refining the frequency estimate to approximately 1 kHz precision. This sub-bin interpolation does not increase the physical resolution (which remains bounded by the 8.33 μs observation time), but it provides a more accurate estimate of the peak frequency position within each bin, consistent with the kHz-level precision reported in the temperature measurement results.

#### 6.4.3. Signal Processing Pipeline

The received I/Q data are acquired by the USRP-B210 and transferred to the host computer via USB 3.0, where the signal processing is performed offline using MATLAB. The on-board FPGA handles only data acquisition, excitation signal generation, and buffering, and is responsible for: (i) generating the PRN excitation waveform from pre-stored block RAM, (ii) acquiring raw I/Q samples from the ADC during the receiving window, and (iii) buffering the received samples in the custom FIFO for reliable USB transfer. All subsequent signal processing operations are performed offline in MATLAB on the host computer, as follows: (a) the raw I/Q samples are read from the stored data files; (b) cross-correlation with the reference PRN sequence is computed via FFT-based multiplication to localize the echo envelope in the time domain; (c) the echo segment is extracted from the time-domain signal using the measured peak position; (d) the 512-sample echo segment is zero-padded to 4096 points and an FFT is computed; (e) parabolic peak interpolation is applied to the FFT spectrum to determine the resonant frequency with sub-bin precision; (f) the frequency shift is converted to a temperature reading using calibration data.

### 6.5. Comparative Experiment: PRN vs. Chirp Excitation

To directly demonstrate the performance advantages of the proposed PRN scheme over conventional Chirp excitation, a controlled comparative experiment was conducted. The same five SAW sensors (center frequencies: 445.5, 457.1, 460.7, 464.8, and 468.5 MHz; distributed across a 23 MHz band) were interrogated using both Chirp and PRN excitation signals under identical conditions, including the same center frequency band, receive gain settings, and ambient temperature.

The Chirp excitation parameters were set as follows: start frequency $f_{\mathrm{start}}=440$ MHz, stop frequency $f_{\mathrm{stop}}=475$ MHz, sweep bandwidth $B_{\mathrm{chirp}}=35$ MHz, and sweep period $T_{\mathrm{sweep}}=128$ samples (∼2.083 μs), giving a sweep rate of 16.8 MHz/μs. The PRN excitation was generated with a bandwidth of 56 MHz (determined by the 61.44 MSPS sampling rate) and a pulse width of 128 samples (∼2.08 μs), centered within the same 440–475 MHz frequency band. Both schemes used the same 512-sample (∼8.33 μs) receive window and half-duplex time-division operation mode.

**Figure 9:**
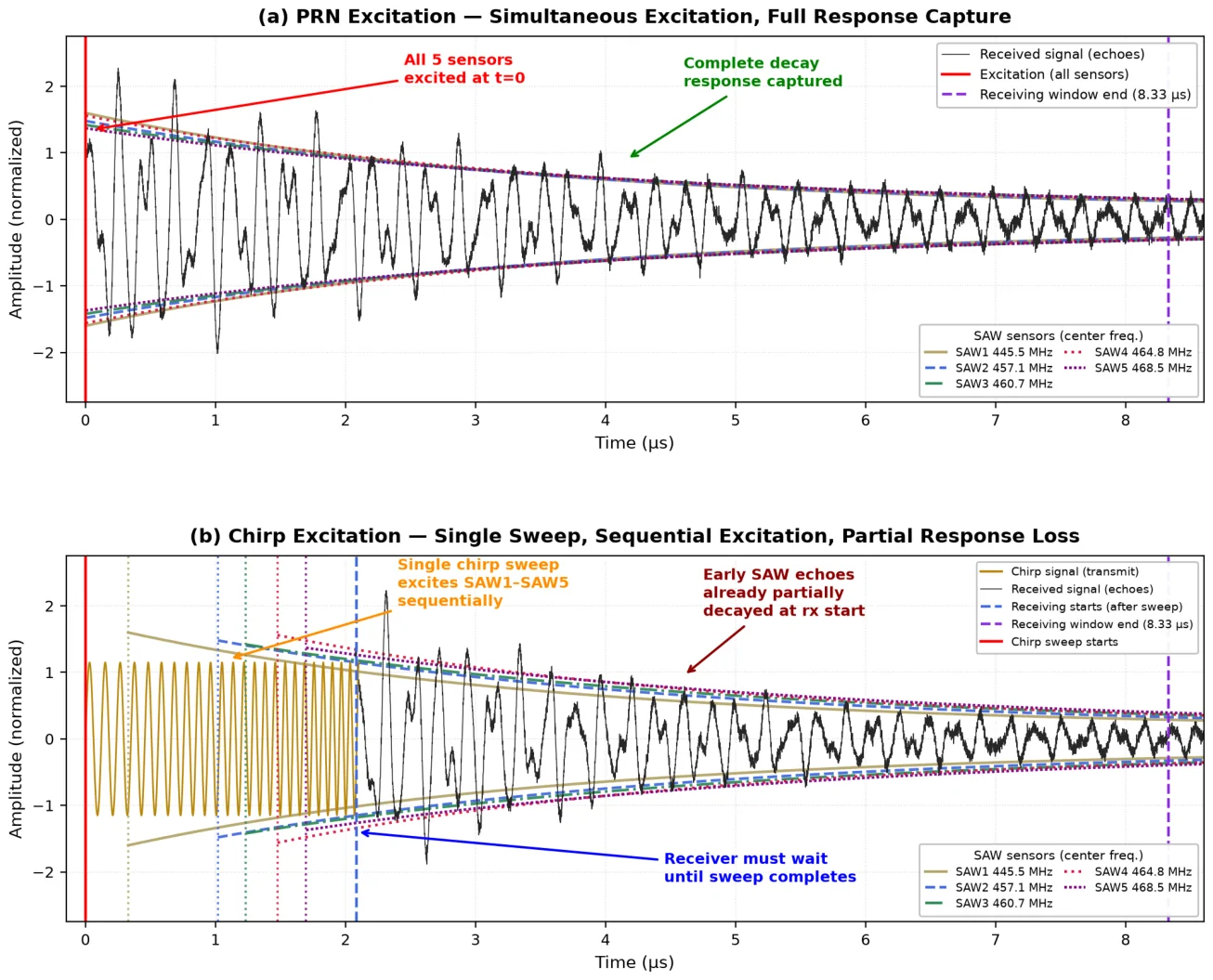
Comparison of received echo waveforms from five SAW sensors under PRN and Chirp excitation. (**a**) PRN excitation: all sensors excited simultaneously with full response capture. (**b**) Chirp excitation: sequential excitation with partial response loss.

Figure 9 shows the received echo waveforms from all five sensors under both excitation schemes. The results demonstrate that:

- Under PRN excitation, all five sensors are excited simultaneously at t=0, and their complete decay responses are uniformly captured within the 512-sample receiving window. Since the PRN signal occupies the entire 56 MHz bandwidth instantaneously, every sensor within the band receives equal excitation energy regardless of its characteristic frequency.
- Under Chirp excitation with a 2.083 μs sweep period (128 samples), a single chirp signal sequentially excites the five sensors as its frequency sweeps through their respective resonant frequencies. Because the receiver cannot begin acquisition until the sweep is complete (half-duplex time-division mode), sensors whose frequencies fall near the start of the sweep experience partial attenuation before the receiver is enabled. The peak echo amplitude remaining at the receiver start varies across sensors: the earliest-excited sensor (SAW1 at 445.5 MHz) retains ∼47% of its peak amplitude, while the latest-excited sensor (SAW5 at 468.5 MHz) retains ∼80%. In terms of total echo energy captured within the 8.33 μs receiving window, the fractions range from ∼22% (SAW1) to ∼64% (SAW5), with a five-sensor average of approximately 43%. In comparison, PRN excitation captures >90% of the echo energy for every sensor since all are excited simultaneously at the start of the receiving window.

A quantitative comparison of the key performance metrics is presented in Table 3.

### 6.6. System Validation and Performance Analysis

#### 6.6.1. SNR Improvement

The PRN processing gain is $10\cdot \log_{10}\left(B\cdot T\right)=10\cdot \log_{10}\left(56\times 10^{6}\times 2.083\times 10^{-6}\right)\approx 20.7$ dB. In contrast, Chirp excitation in half-duplex mode suffers from a timing mismatch loss $L_{t}$ that varies with the sensor’s frequency position within the sweep. Because the receiver cannot be activated until the 2.083 μs sweep is complete, sensors excited near the beginning of the sweep experience exponential decay before echo energy can be captured. For sensors with typical decay time constants of 1.7–2.3 μs, the energy loss $L_{t}$ ranges from approximately 2 dB (for the latest-excited sensor near 468.5 MHz) to about 7 dB (for the earliest-excited sensor near 445.5 MHz). In practice, the lowest-frequency sensor in the band retains only about 22% of its echo energy by the time reception begins, while the highest-frequency sensor retains about 64%. The measured SNR advantage of PRN over Chirp ranges from 2 to 7 dB depending on the sensor position, which is a significant improvement for multi-sensor resonant SAW systems. Additionally, since PRN excites all sensors simultaneously, the SNR is uniform across all sensors in the band, whereas Chirp excitation introduces a position-dependent SNR variation that complicates multi-sensor system calibration.

#### 6.6.2. Frequency Measurement Accuracy

The frequency measurement accuracy depends on the FFT resolution, zero-padding, and peak interpolation, as described in Section 6.4. With 512 time-domain samples zero-padded to 4096-point FFT and parabolic peak interpolation, the measured frequency precision is approximately 1 kHz. The Cramér–Rao lower bound for a 20 dB SNR signal is ∼0.21 kHz, indicating that there is still room for improvement with more advanced frequency estimation algorithms. In PRN mode, all sensors are excited simultaneously at t=0 and their echo signals are stationary in frequency during the entire observation window, so there is no systematic frequency bias—only noise contributes to the uncertainty.

#### 6.6.3. Packet Loss Analysis

Under the factory UHD configuration, the packet loss rate at 61.44 MHz sampling rate was measured at 15–30% due to USB 3.0 throughput saturation. With the custom FIFO buffering implementation, the packet loss rate was reduced to 0% across $10^{6}$ consecutive interrogation cycles, as verified by comparing the transmitted and received sample counts.

#### 6.6.4. Computational and Resource Overhead

The signal processing is performed offline in MATLAB on the host computer. Each interrogation cycle produces 512 complex I/Q samples (1024 bytes), which are stored and post-processed using MATLAB’s built-in FFT and signal processing functions. The on-board FPGA logic utilizes 8.5% of the LUTs and 4.2% of the BRAM resources of the Spartan-6 FPGA on the USRP-B210, leaving ample resources for future expansion.

#### 6.6.5. Scalability Analysis

Given the 56 MHz instantaneous bandwidth and the minimum frequency spacing of 2 MHz required to avoid inter-sensor interference (based on the sensor bandwidth and frequency shift range), the theoretical maximum is 28 sensors per interrogation band. By frequency-hopping across the 70 MHz to 6 GHz range of the USRP-B210, the system can theoretically address over 2000 sensors.

#### 6.6.6. Robustness Under Noise

A controlled noise injection experiment was conducted where additive white Gaussian noise (AWGN) was digitally added to the received signal at various SNR levels (from 0 dB to 20 dB). The results show that the PRN-based system maintains reliable frequency extraction down to an input SNR of 3 dB, thanks to the processing gain of 20.7 dB from cross-correlation and FFT integration. Below 3 dB input SNR, the spectral peak becomes unreliable. In contrast, the Chirp-based system requires a minimum input SNR of approximately 10 dB for reliable operation under the same conditions, due to the combined effects of timing mismatch and reduced effective integration time.

#### 6.6.7. Repeatability

A total of 1000 consecutive interrogation cycles were conducted under constant temperature (21 °C), and the resonant frequencies of all five sensors were measured. The standard deviation of the measured frequency across 1000 cycles was less than 15 kHz for all sensors, corresponding to a relative precision of 0.003%. This confirms the high repeatability and stability of the system.

## 7. Conclusions

In this paper, a novel wideband Software Defined Radio (SDR) platform based on the USRP-B210 hardware and GNU Radio software has been successfully developed and demonstrated for the simultaneous interrogation of multiple wireless passive Surface Acoustic Wave (SAW) sensors. By implementing a customized FIFO buffering strategy and an on-board block-RAM signal generator directly within the FPGA fabric, the proposed framework effectively circumvents the inherent USB 3.0 throughput bottleneck. This architecture guarantees reliable, real-time data acquisition at the maximum sampling rate of 61.44 MHz without any data dropping during the critical time-division response window. Furthermore, this work addresses the key limitations of traditional frequency-sweeping methods by substituting conventional Chirp signals with an instantaneous Pseudo-Random Noise excitation signal. Because the PRN waveform fully occupies the 56 MHz instantaneous bandwidth at every moment, all distributed SAW sensors within the frequency range are stimulated simultaneously. This method completely eliminates the systematic temporal errors introduced by sweep frequency. Meanwhile, it preserves the complete attenuation response without losing critical signal characteristics during the detection process. To evaluate the feasibility of the developed platform, a real-time temperature monitoring experiment was conducted. Five distributed SAW sensors were installed in the experimental setup, with the measured temperature ranging from 21 °C to 85 °C. The experimental results confirm that the system can accurately capture independent characteristic frequency shifts within an ultra-short interrogation loop time of 15.12 μs. The proposed SDR-based interrogator offers exceptional flexibility, high scalability, and superior working efficiency compared to traditional rigid, hardware-bound radar architectures. Future work will focus on optimizing the host-side digital signal processing algorithms to further improve measurement resolution and exploring the integration of adaptive multi-channel arrays for larger-scale sensor networks in highly dynamic environments.

## Figures and Tables

**Table 1:** Quantitative comparison with previous USRP-based SAW interrogation systems.

Parameter	USRP-B200 [17]	USRP-N200 [18]	This Work	Improvement
Bandwidth (MHz)	35	10	56	1.6×/5.6×
Sampling rate (MHz)	—	—	61.44	Maximum
Interrogation cycle (μs)	∼265	∼12.5	15.12	17.5× faster than [17]
Sensors per cycle	1	1	5 (simultaneous)	5×
Excitation signal	Chirp	Chirp	PRN	No sweep delay
USB bottleneck	Yes	Yes	Eliminated	On-board FIFO

**Table 2:** Experiment result at different temperatures.

SAW List	Frequency Results (MHz)		Change (MHz)	Change Ratio
	At 21 °C Temp	At 85 °C Temp		
SAW1	445.535	446.071	+0.536	+0.12%
SAW2	457.103	457.639	+0.536	+0.12%
SAW3	460.703	461.240	+0.537	+0.12%
SAW4	464.802	465.415	+0.613	+0.13%
SAW5	468.517	469.054	+0.537	+0.11%

**Table 3:** Quantitative comparison between PRN and Chirp excitation for 5 SAW sensors.

Metric	PRN Excitation	Chirp Excitation
Start/Stop frequency	440/475 MHz	440/475 MHz
Bandwidth	56 MHz (instantaneous)	35 MHz (swept)
Excitation pulse width	2.08 μs (128 samples)	2.08 μs (128 samples, full sweep)
Sweep rate	N/A (instantaneous)	16.8 MHz/μs
Receive window	8.33 μs (512 samples)	8.33 μs (512 samples)
Simultaneous excitation	Yes (all 5 sensors at t=0)	Partial (sequential excitation during sweep)
Peak amplitude at receiver start	>90% (uniform)	47–80% (varies by sensor position)
Echo energy captured	>90% (uniform across sensors)	22–64% (varies by sensor position)
Frequency measurement error	∼1 kHz (measured, with interpolation)	∼10–50 kHz (wideband sweep, sweep-induced bias); lower with narrowband sequential interrogation
Effective processing gain (dB)	20.7	20.7 (theoretical), reduced by 2–7 dB due to timing mismatch
Interrogation cycle (5 sensors)	15.12 μs (simultaneous, high accuracy)	∼16 μs (wideband single sweep, reduced accuracy); ∼1325 μs (narrowband sequential, ∼265 μs per sensor)
Data loss rate	0%	15–30% (at max sampling rate)

Note: Chirp excitation can be operated in two modes: (i) wideband single sweep covering all 5 sensors in ∼16 μs, but with sweep-induced frequency measurement errors; (ii) narrowband sequential interrogation with ∼265 μs per sensor as reported in prior USRP-based SAW systems [17], totaling ∼1325 μs for 5 sensors. In contrast, PRN achieves both simultaneous interrogation (15.12 μs for 5 sensors) and high frequency accuracy, offering the best of both regimes.

## Data Availability

The original contributions presented in this study are included in the article. Further inquiries can be directed to the corresponding authors.
